# Overexpression of Hsp104 by Causing Dissolution of the Prion Seeds Cures the Yeast [*PSI^+^*] Prion

**DOI:** 10.3390/ijms241310833

**Published:** 2023-06-29

**Authors:** Katherine E. Stanford, Xiaohong Zhao, Nathan Kim, Daniel C. Masison, Lois E. Greene

**Affiliations:** 1 Laboratory of Cell Biology, National Heart, Lung and Blood Institute, National Institutes of Health, Bethesda, MD 20892, USA; 2 Laboratory of Biochemistry and Genetics, National Institute of Diabetes and Digestive and Kidney Diseases, National Institutes of Health, Bethesda, MD 20892, USA

**Keywords:** prion, yeast, curing, Hsp104, overexpression, trimming

## Abstract

The yeast Sup35 protein misfolds into the infectious [*PSI^+^*] prion, which is then propagated by the severing activity of the molecular chaperone, Hsp104. Unlike other yeast prions, this prion is unique in that it is efficiently cured by the overexpression as well as the inactivation of Hsp104. However, it is controversial whether curing by overexpression is due to the dissolution of the prion seeds by the trimming activity of Hsp104 or the asymmetric segregation of the prion seeds between mother and daughter cells which requires cell division. To answer this question, we conducted experiments and found no difference in the extent of curing between mother and daughter cells when half of the cells were cured by Hsp104 overexpression in one generation. Furthermore, curing was not affected by the lack of Sir2 expression, which was reported to be required for asymmetric segregation of the [*PSI^+^*] seeds. More importantly, when either hydroxyurea or ethanol were used to inhibit cell division, the extent of curing by Hsp104 overexpression was not significantly reduced. Therefore, the curing of [*PSI^+^*] by Hsp104 overexpression is not due to asymmetric segregation of the prion seeds, but rather their dissolution by Hsp104.

## 1. Introduction

Prion proteins have a properly folded conformation and an infectious misfolded conformation rich in beta-sheet [1,2,3]. They are not only present in mammals [4], but also in *Saccharomyces cerevisiae*, which has made yeast a model organism to study prion transmission [3,5,6]. In yeast, the misfolded conformation exists as prion seeds that are transmitted from mother to daughter cells upon cell division or during mating [5,7,8]. The occurrence of many of the yeast prions is dependent on the molecular chaperone, Hsp104, which severs the prion seeds in an ATP-dependent reaction, thereby maintaining the steady-state number of seeds as the yeast divides [9,10,11,12,13,14,15]. When the Hsp104 ATPase activity is inactivated, the severing of the seeds is inhibited, causing them to be diluted by cell division [10,13,16]. Then, once all of the seeds are eliminated, the yeasts are then cured of prion with the misfolded amyloid conformation [12,17].

We have been studying the [*PSI^+^*] yeast prion, which forms when the translation termination protein, Sup35, misfolds into amyloid fibers or prion seeds [5,18]. Since this prion must be severed by Hsp104, as expected, the inactivation of Hsp104 cures the yeast of the [*PSI^+^*] prion [11,12,13]. However, in contrast to other yeast prions, overexpression of Hsp104 efficiently cures the [*PSI^+^*] prion [14]. It is known that the kinetics of curing caused by the inactivation of Hsp104 is very different from the kinetics of curing caused by Hsp104 overexpression [19,20,21]. Unlike curing by Hsp104 overexpression, when Hsp104 is inactivated, the kinetics show a lag during which the seeds are diluted by cell division [12,13,17]. The yeasts are only cured after all of the seeds have been eliminated [17].

Like other yeast prions, [*PSI^+^*] prion occurs as different variants. Structural studies have shown that the different [*PSI^+^*] variants differ in their conformations and biophysical properties [22,23,24,25]. The weak [*PSI^+^*] variants, which have fewer seeds and more soluble Sup35 than the strong [*PSI^+^*] variants, are cured faster by Hsp104 overexpression than the strong [*PSI^+^*] variants [18,26]. Furthermore, low levels of Hsp104 overexpression can cure the weak [*PSI^+^*] variants, but not the strong [*PSI^+^*] variants [26]. 

Although the paradoxical finding that overexpression of Hsp104 cures [*PSI^+^*] was discovered more than 25 years ago, there is still no consensus on the mechanism of this curing. An understanding of the mechanism is confounded by the finding that other proteins, such as Hsp90 and the cochaperone STI1, also affect the rate of [*PSI^+^*] curing by Hsp104 overexpression [27]. Several models have been proposed to explain the curing of [*PSI^+^*] by Hsp104 overexpression. One proposed mechanism is that this curing is caused by asymmetric segregation of the prion seeds [20,28]. In this model, instead of the seeds freely diffusing through the bud necks from mother cells to daughter cell, the seeds are retained in the mother cell, which leads to faster curing of the daughter cells than the mother cells. Originally, evidence for this model came from running yeast lysates on semi-denaturing gels, which showed that when Hsp104 was overexpressed, there was an increase in the size of the partially depolymerized [*PSI^+^*] seeds [29]. However, a recent study from the Tuite laboratory did not find such a difference in seed size even though in a minority of the cells, the number of prion seeds in the mother cell was greater than in the daughter cell [20]. They therefore suggested that the difference in retention was due to the seeds being anchored to a subcellular structure in the mother cell. 

Another model proposed by the Mogk laboratory is that when Hsp104 is overexpressed, it causes curing by binding to the seeds in a nonproductive conformation that inhibits the severing of the seeds [30]. However, the kinetics of curing does not fit with this proposed model of curing [19,20]. Specifically, the kinetics of curing by Hsp104 overexpression does not show a lag prior to the onset of curing [19,20,21], which would have to occur if the curing was caused by Hsp104 inactivation.

A third model of curing is that overexpression of Hsp104 causes dissolution of the prion seeds; we proposed this model based on our imaging of the GFP-labeled Sup35 foci when Hsp104 was overexpressed [19]. We previously observed that Hsp104 has two different activities: severing activity that makes the seeds smaller and increases their number and trimming activity that also makes the seeds smaller but does not change their number [31]. We observed that, when Hsp104 is overexpressed, there is a loss of detectable foci prior to the curing of the [*PSI^+^*] prion, which suggested that this curing is caused by an increase in the trimming of the prion seeds by Hsp104 [19,26]. Further support for the dissolution model came from separating mother from daughter cells or old from young cells [19]. Separation of these different populations showed no difference in the rate of curing as would be expected if curing was caused by asymmetric segregation. 

There are two key differences between the asymmetric segregation model and the dissolution model for the curing of [*PSI^+^*] by Hsp104 overexpression. One prediction is that, if curing is caused by asymmetric segregation of the seeds, then the daughter cells should cure faster than the mother cells, whereas if curing is caused by dissolution of the seeds, mother and daughter cells should cure at the same rate. Another key difference between these models is that curing by asymmetric segregation has been reported to require Sir2 [28] and more importantly, definitely requires cell division, whereas curing by dissolution does not require any of these mechanisms. 

Here, we have reexamined the curing of [*PSI^+^*] by Hsp104 overexpression. First, using a weak [*PSI^+^*] variant, we showed that mother and daughter cells cure at the same rate, consistent with our previous observations [19]. Second, we found that inhibiting cell division with either hydroxyurea or ethanol did not significantly affect the curing of a weak [*PSI^+^*] variant. In fact, we were able to almost completely block cell division and still cure [*PSI^+^*] by Hsp104 overexpression in 10% ethanol nor did curing by Hsp104 overexpression require Sir2. Interestingly, ethanol, which both arrests cell division and induces higher levels of Hsp104 [32], was sufficient to cure [*PSI^+^*] without having to overexpress Hsp104 from a plasmid. These data show that [*PSI^+^*] can be cured without cell division and therefore, strongly support the dissolution model of curing by Hsp104 overexpression.

## 2. Results

### 2.1. Curing of [PSI^+^] by Hsp104 Overexpression

**Figure 1:**
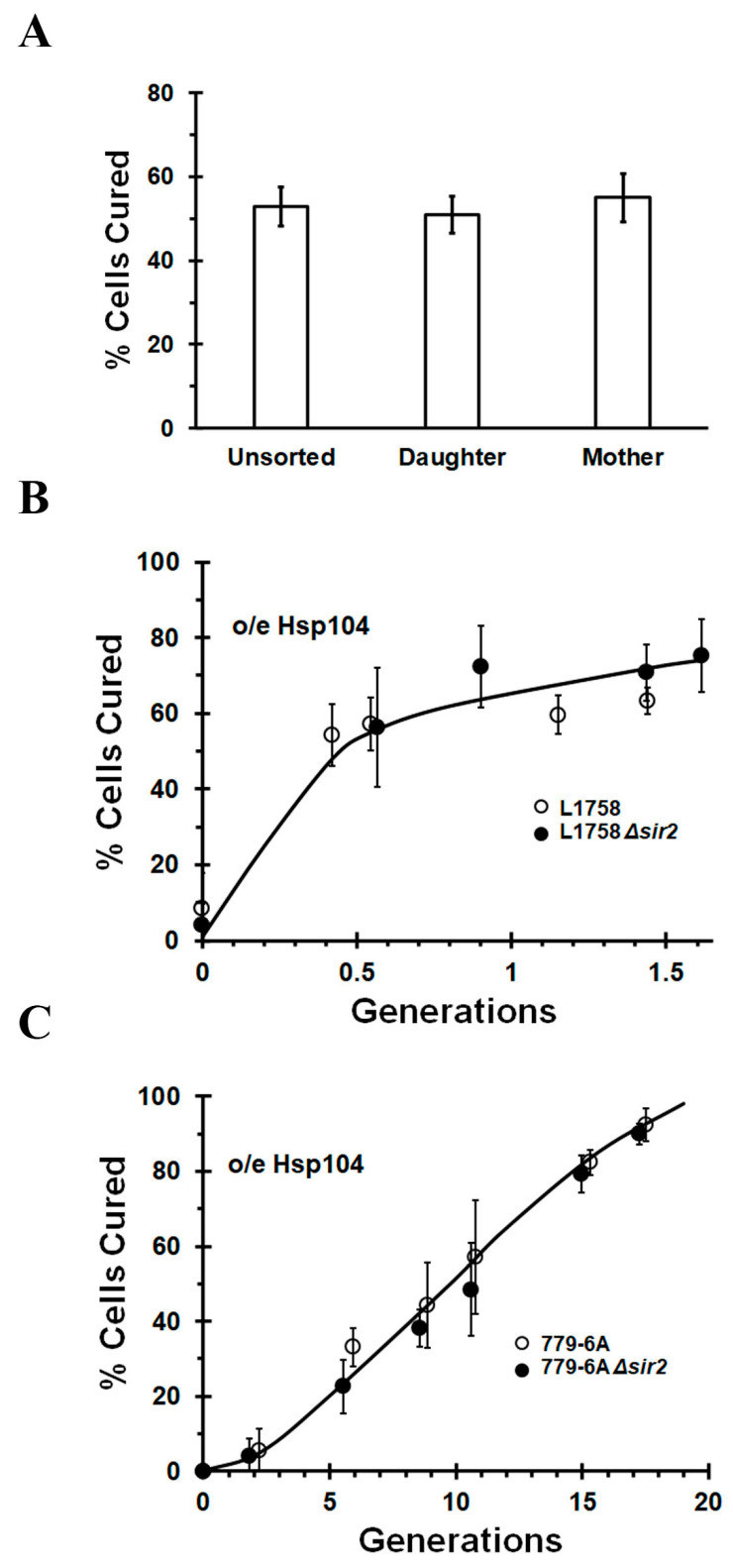
The curing of [*PSI^+^*] by Hsp104 overexpression shows that the mother and daughter cells cure at the same rate and this rate of curing is not affected by Sir2 expression. (**A**) Fluorescently labeled mother cells and non-labeled daughter cells were separated via flow cytometry after overexpressing Hsp104 for one generation in the L1758 [*PSI^+^*] strain. The percentage of curing in these two separated cell populations was then measured using the red/white plating assay. (**B**) The L1758 and L1758 Δ*sir2* [*PSI^+^*] strains cure at the same rate when Hsp104 was overexpressed from the *GAL1* promoter (**C**) The 779-6A and 779-6A Δ*sir2* [*PSI^+^*] strains cure at the same rate when Hsp104 was overexpressed from the *GAL1* promoter. The 779-6A strain contains a much stronger [*PSI^+^*] variant than the L1758 strain.

There are two features that distinguish the asymmetric segregation model of [*PSI^+^*] curing by Hsp104 overexpression from the dissolution model of curing. One feature is the greater retention of seeds in the mother than the daughter cell, which, in turn, leads to faster curing of the daughter than the mother cell [21]. We first reexamined whether we could detect a difference in the rate of curing between mother and daughter cells using the weak L1758 [*PSI^+^*] strain instead of the stronger [*PSI^+^*] variant that we used in our previous study [19]. Instead of the ~15% curing per generation that we obtained when the strong [*PSI^+^*] variant was cured by Hsp104 overexpression, we obtained ~50% curing per generation when the weak L1758 [*PSI^+^*] variant was used (Figure 1A). This increased the accuracy of our measurement of the difference between the curing of mother versus daughter cells.

The L1758 [*PSI^+^*] strain was transformed with a centromeric plasmid to overexpress Hsp104 from the *GAL1* promoter. Following overnight growth in raffinose medium, the yeast wall was labeled with a fluorescent tag. Galactose was then added to induce Hsp104 overexpression, and the yeast were grown for 4 h (~1 generation) prior to separating the fluorescent mother cells from the non-fluorescent daughter cells via flow cytometry. These two populations were then plated on ½ YPD plates and the extent of curing was measured using the red/white colony assay. In this assay, only completely red colonies as opposed to red/white sectored colonies indicate cured cells. As shown in Figure 1A, there was no significant difference in the extent of curing of the mother and daughter cells; i.e., both populations were ~50% cured. Since there was not a significant difference in the curing of the mother and daughter cells, the mother cells did not retain more seeds than the daughter cells. Therefore, consistent with our previous data [19], we find no evidence for asymmetric segregation of the prion seeds during the curing of [*PSI^+^*] by Hsp104 overexpression. 

We next examined whether deletion of *SIR2* affected the curing of the L1758 [*PSI^+^*] strain. Sir2 is a protein that is required for the formation of the polarisome, which, in turn, functions in the transport of misfolded proteins from daughter to mother cell [33,34], and therefore might play a role in asymmetric segregation of the prion seeds. Based on our flow cytometry results, we expected that the rate of curing of L1758 [*PSI^+^*] by Hsp104 overexpression would not be affected by Sir2 expression. As shown in Figure 1B, this is indeed the case. There was no significant difference in the rate of curing whether or not Sir2 was expressed. Similar results were obtained using another yeast strain, 779-6A, which contains a stronger [*PSI^+^*] variant. As with the L1758 [*PSI^+^*] strain, the curing of the 779-6A [*PSI^+^*] strain by Hsp104 overexpression was not affected by Sir2 expression (Figure 1C). Therefore, these results further support our conclusion that the curing of [*PSI^+^*] by Hsp104 overexpression is not due to asymmetric segregation of the prion seeds.

### 2.2. Curing of [PSI^+^] by Hsp104 Overexpression in 10% Ethanol

**Figure 2:**
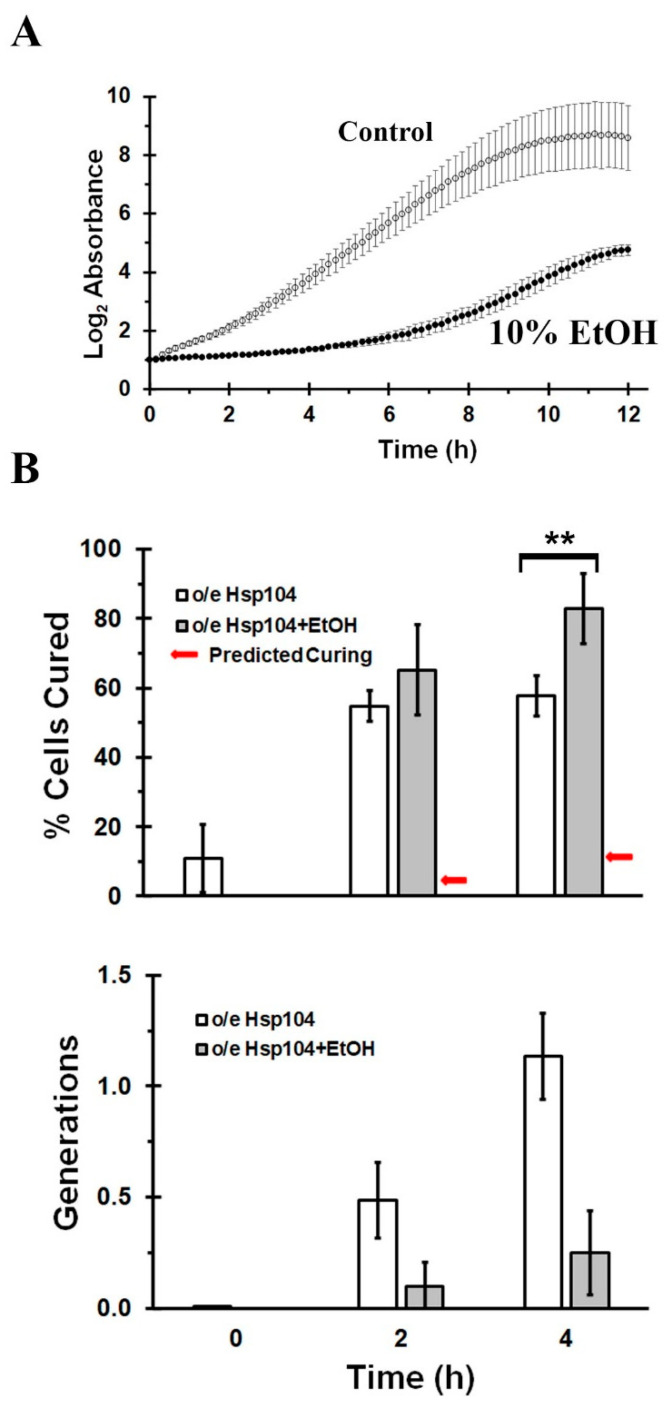
Effect of 10% ethanol on yeast growth and the curing of [*PSI^+^*] by Hsp104 overexpression. (**A**) The growth of L1758 [*PSI^+^*] strain in 10% ethanol when assayed at 600 nm on a plate reader. (**B**) The curing of the L1758 [*PSI^+^*] strain by Hsp104 overexpression was measured in the presence and absence of 10% ethanol after 2 h or 4 h in synthetic galactose medium. In the top panel, the percent cured cells are plotted as a function of incubation time and in the bottom panel, the generations are plotted as a function of incubation time. The red arrow indicates the predicted maximum value for the percent cured cells based on the asymmetric model of curing based on the number of generations measured in the lower panel. ** indicates *p* ≤ 0.01.

The second feature that distinguishes the asymmetric segregation model from the dissolution model is that, in the former but not the latter model, cell division is required for the curing of [*PSI^+^*] by Hsp104 overexpression [19]. This prompted us to examine whether we could cure [*PSI^+^*] when cell division was arrested by incubating the yeast in 10% ethanol [32,35,36]. When yeast growth was measured, the graph of absorbance versus time (Figure 2A) showed that 10% ethanol arrested cell growth for about 6 h and then with further incubation time, the yeast again started to divide. We also tested yeast viability in ethanol by plating the yeast on YPD plates both prior to addition of ethanol and after 4 h in 10% ethanol. From the colony count, we found that ethanol caused only a 10% reduction in yeast viability. Based on these results, our experiments were conducted in 10% ethanol over a 4 h time period. 

We next measured the curing of the L1758 [*PSI^+^*] strain by overexpressing Hsp104 from the *GAL1* promoter in 10% ethanol, which arrests cell division. If the curing of [*PSI^+^*] is due to asymmetric segregation of the prion seeds, at most 50% curing in one generation would occur if all of the seeds were retained in the mother cells and none pass to the daughter cells when the yeast divide. More generally, the maximum percentage of curing that can occur by the asymmetric segregation model is 50% of the generation time. Therefore, extensive curing with minimum yeast growth is not consistent with the asymmetric segregation model of curing. 

To test this model, the L1758 [*PSI^+^*] yeast strain was grown in galactose both in the presence and absence of ethanol. After 2 h and 4 h, we measured the curing using the red/white colony assay and the growth by measuring the absorbance at 600 nm. Figure 2B shows the percentage of cured cells (top panel) and the growth in generations (bottom panel), which were both plotted as a function of time. These data show that inhibiting cell division by addition of 10% ethanol does not prevent curing by Hsp104 overexpression. 

As shown in Figure 2, the yeast grew 0.5 generations in the absence of ethanol and 0.1 generations in the presence of ethanol in 2 h in galactose medium, while in both cases, the yeast were ~55% cured. After 4 h in galactose medium with no ethanol, the yeast were ~65% cured in 1.1 generations, whereas in the presence of ethanol the yeast were ~85% cured in only 0.25 generations. These results show that even in the absence of ethanol, we observed more curing than predicted based on the asymmetric segregation model of curing. When Hsp104 was overexpressed for 2 h, there was ~50% curing of L1758 [*PSI^+^*] in 0.5 generations, whereas the asymmetric segregation model predicts only 25% curing if all of the seeds were retained in the mother cell. The large discrepancies between the predicted and measured values for the percent cured based on the asymmetric segregation model is particularly obvious in the presence of ethanol when cell division is blocked. The red arrows in Figure 2B show the maximum amount of curing in ethanol calculated from the measured generation times according to the asymmetric segregation model. Specifically, at 0.1 and 0.25 generations, ~55% and ~85% of the L1758 [*PSI^+^*] yeast were cured, respectively, while the predicted values for the percentages cured based on the asymmetric segregation model are 5% and 12%, respectively. Therefore, by comparing the observed curing with the maximum curing that could occur based on the asymmetric segregation model, we observe an order of magnitude more curing than predicted.

### 2.3. Curing of [PSI^+^] in 10% Ethanol Alone

Interestingly, we obtained significantly more curing of the L1758 [*PSI^+^*] strain by Hsp104 overexpression when the yeast were incubated in galactose for 4 h in the presence of ethanol than in its absence, which indicates that ethanol is contributing to the curing of this [*PSI^+^*] strain. This suggests that 10% ethanol alone might also cure this [*PSI^+^*] strain without expressing Hsp104 from a plasmid, particularly because the stress caused by ethanol has itself been shown to increase Hsp104 expression [37]. In fact, 10% ethanol was shown to cause loss of the [*PSI^+^*] phenotype by the Cox laboratory [38] before [*PSI^+^*] was even recognized to be a prion. However, the Tuite laboratory found that ethanol only transiently increased the translation termination of [*PSI^+^*] yeast since even after washing out the ethanol, the yeast were still [*PSI^+^*] [39].

**Figure 3:**
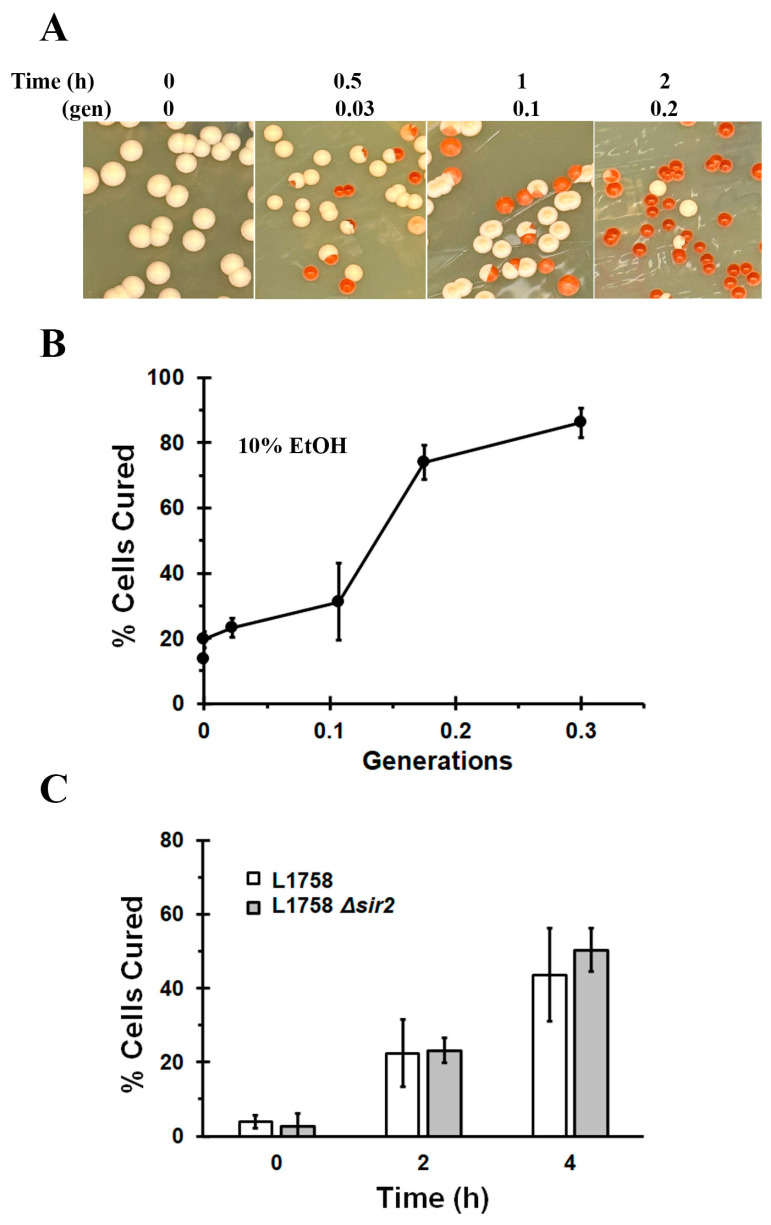
The curing of L1758 [*PSI^+^*] in 10% ethanol. (**A**) The L1758 cells transformed with pRS314 empty vector in synthetic galactose medium was incubated in 10% ethanol. At different times, the yeasts were plated on the ½ YPD plates. Only completely red colonies are counted as cured and either white or white/red colonies are counted as [*PSI^+^*]. The time is given both in terms of hours (h) and generations (gen). (**B**) The data from the red/white colony assay were graphed as % cured vs. generations. (**C**) The L1758 [*PSI^+^*] and the L1758 Δ*sir2* [*PSI^+^*] yeast cured at the same rate in 10% ethanol. The L1758 [*PSI^+^*] and L1758 [*PSI^+^*] Δ*sir2* strains transformed with empty vector were incubated in synthetic defined medium with glucose and 10% ethanol for the indicated times.

To test whether addition of 10% ethanol alone cures the L1758 [*PSI^+^*] strain, this strain was transformed with an empty vector rather than with a plasmid that overexpresses Hsp104. Ethanol and galactose were then added to the transformed strain, followed by measuring both the prion curing and growth at different times. The ½ YPD plates in Figure 3A show a progressive increase in red colonies as a function of incubation time, which indicates that ethanol alone cured the L1758 [*PSI^+^*] strain. From the red/white colony assay and the absorbance, we plotted the data as percent cured cells versus generation time (Figure 3B). After 30 min in ethanol (0.03 generations), ~25% of the cells were cured and the percentage cured continue to increase over the next 2 h so that ~75% of the yeast were cured after the yeast grew only 0.2 generations. Finally, after 4 h in 10% ethanol the curing increased to ~85% while absorbance measurements showed that the yeast grew only 0.3 generations. Therefore, 10% ethanol was sufficient to cure the L1758 [*PSI^+^*] strain in a raffinose/galactose medium.

Since yeast prefer glucose rather than raffinose/galactose as a carbon source and the carbon source has been shown to affect Hsp104 expression [40], we next examined whether the L1758 [*PSI^+^*] strain was cured in 10% ethanol with glucose instead of raffinose/galactose in the selection medium. We found that, after 4 h (0.3 generations) in 10% ethanol, ~50% of the L1758 [*PSI^+^*] was cured in synthetic medium with glucose as a carbon source (Figure 3C), which shows that curing in 10% ethanol occurred with different fermentable carbon sources. These results further support our observation that [*PSI^+^*] is cured in the absence of cell division in 10% ethanol. 

Since the curing of the L1758 [*PSI^+^*] strain is independent of cell division in 10% ethanol, this curing should also not be affected by Sir2 expression. As noted above, Sir2 functions in the formation of the polarisome, which transports misfolded proteins from daughter to mother cells. The effect of Sir2 expression was tested by measuring the curing of the L1758 [*PSI^+^*] Δ*sir2* strain in 10% ethanol and glucose medium. As shown in Figure 3C, the L1758 [*PSI^+^*] strain was ~50% cured when the yeast were incubated in 10% ethanol for 4 h regardless of Sir2 expression. These results further support the conclusion that the curing of L1758 [*PSI^+^*] in 10% ethanol is not due to asymmetric segregation of the seeds. 

**Figure 4:**
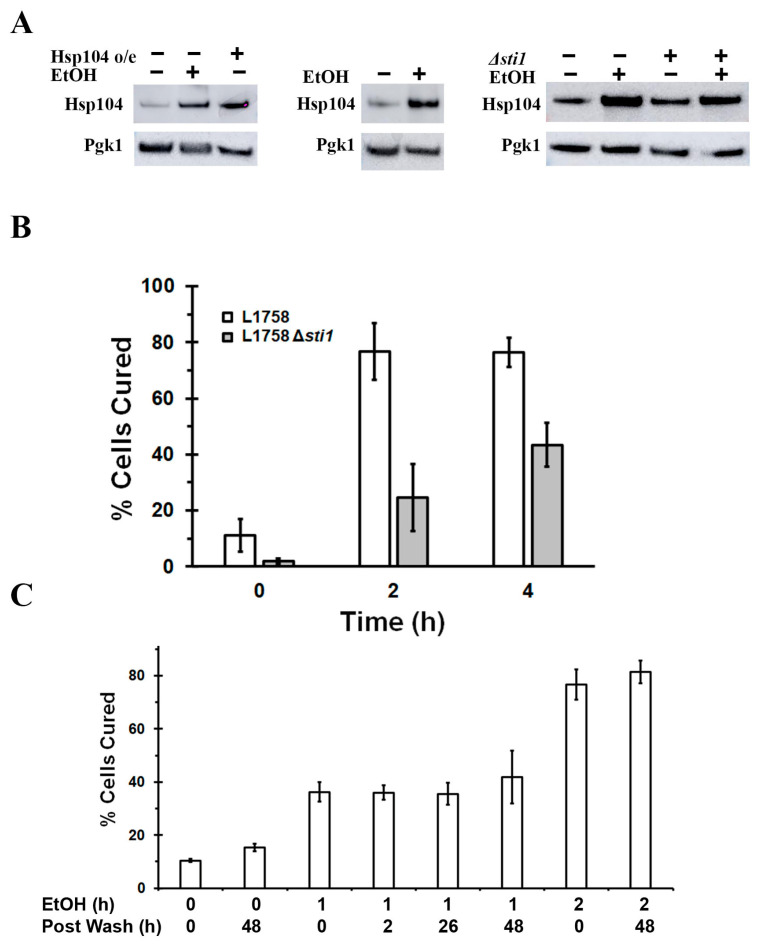
Ethanol induces Hsp104 expression, which, in turn induces curing of the L1758 [*PSI^+^*] strain. (**A**) Western blots of Hsp104 induction by ethanol under different conditions. In the left panel, L1758 [*PSI^+^*] strain was either transformed with empty vector or a vector to express Hsp104 from the *GAL1* promoter. The yeast transformed with the vector control in galactose medium were incubated in 10% ethanol for 2 h, whereas the yeast with the Hsp104 overexpression plasmid was incubated in galactose medium for 2 h. In the middle panel, yeast transformed with the vector control was incubated in selection medium for 2 h in the presence and absence of 10% ethanol. In the right panel, the Hsp104 level in the L1758 and the L1758 Δ*sti1* [*PSI^+^*] strains transformed with empty vector were incubated in synthetic galactose medium in the presence and absence of 10% ethanol for 2 h. (**B**) Effect of Sti1 expression on the curing of [*PSI^+^*] in 10% ethanol. The yeast were incubated in ethanol for either 2 or 4 h. (**C**) Assay to measure whether the curing of [*PSI^+^*] occurs after plating on YPD plates. L1758 [*PSI^+^*] strain was incubated in synthetic galactose medium in 10% ethanol for either 1 h or 2 h. After plating the yeast, the remaining yeasts were spun down and then resuspended in a YPD medium. They were then maintained in log phase for 2 days. The yeasts were plated at the indicated times to measure the curing using the red/white colony assay.

To confirm that the expression level of Hsp104 in 10% ethanol is sufficient to cure this strain, we ran a Western blot to compare the level of Hsp104 in a lysate from yeast grown in 10% ethanol for 2 h with the level of Hsp104 in a lysate from yeast overexpressing Hsp104 from the *GAL1* promoter for 2 h. Both of these conditions, which produced >50% curing of the L1758 [*PSI^+^*] strain, increased the expression of Hsp104 when compared to a control to which only raffinose was added (Figure 4A, left panel). When the Hsp104 levels were normalized using the PGK1 loading control, there was a ~3-fold increase in the level of Hsp104 in both the ethanol-treated cells and in the cells overexpressing Hsp104 from the *GAL1* promoter. Similarly, the Hsp104 level was markedly increased when the cells were incubated for 4 h in 10% ethanol and synthetic defined medium with glucose as a carbon source (Figure 4A, middle panel). Taken together, these results show that ethanol increased the level of Hsp104 expressed from the *HSP104* promoter both in glucose and galactose media. Since the expression of Hsp104 induced by 10% ethanol is comparable to the expression of Hsp104 from the *GAL1* promoter at early times after addition of galactose, this shows that the level of expression of Hsp104 induced by ethanol is sufficient to cure the weak L1758 [*PSI^+^*] strain. 

Next, we tested whether the curing of the L1758 [*PSI^+^*] strain in 10% ethanol is really due to the observed increase in Hsp104 expression and not the expression of some other proteins induced by the 10% ethanol by examining the effect of Sti1 expression on curing. Genomic deletion of the cochaperone *STI1* has been previously shown to reduce the curing of [*PSI^+^*] caused by Hsp104 overexpression by reducing Hsp104 activity [27,41]. Therefore, if the curing of the L1758 [*PSI^+^*] strain in 10% ethanol were due to Hsp104, then curing should be reduced when *STI1* is deleted. Indeed, we found that in the absence of Sti1 expression, there was only 30% curing of the L1758 [*PSI^+^*] strain after 4 h in ethanol, a 50% decrease in the extent of curing compared to the parental strain (Figure 4B). To make certain that this decrease in curing was caused by a reduction in Hsp104 activity and not Hsp104 level, we ran a Western blot of yeast lysates to determine the level of Hsp104 in the *STI1* deletion strain. As shown (Figure 4A, right panel), deleting *STI1* did not significantly affect Hsp104 expression either in the presence or absence of ethanol. Therefore, *STI1* is only affecting the activity of Hsp104, which, in turn, confirms that the curing of L1758 [*PSI^+^*] caused by 10% ethanol is due to increased expression of Hsp104. 

Our conclusion that the curing of [*PSI^+^*] in 10% ethanol is independent of cell division is based on the red/white colony plating assay. However, rather than all of the curing occurring before the yeast is plated, there may be curing occurring on the plate until the high intracellular Hsp104 levels are diluted by cell division and degradation. To test whether curing occurred on the plate, we incubated the L1758 [*PSI^+^*] strain in 10% ethanol for either 1 h or 2 h, which when plated, showed that the yeast were 40% and 80% cured, respectively (Figure 4C). The 80% provides an upper limit for the amount of curing that can occur. To examine whether the 40% cured cells approached this value, an aliquot of the ethanol-treated yeast were spun down and then suspended in a YPAD medium. The yeast were then maintained in log phase for another 2 days during which time, they were periodically plated to monitor the curing. If curing did not occur after the yeasts were plated, then there should also be no further curing while the yeasts were growing in the YPAD medium. As shown in Figure 4C, this was in fact the case, suggesting that there was no significant curing after the yeasts were plated. 

### 2.4. Imaging of GFP-Labeled Sup35 during Curing of [PSI^+^] by Hsp104 Overexpression in 10% Ethanol

Next, we imaged GFP-labeled Sup35 in [*PSI^+^*] cells incubated in 10% ethanol to both prevent cell division and induce Hsp104 expression, thus causing curing. Importantly, the asymmetric segregation model and the dissolution model make very different predictions in regard to the changes in GFP-labeled Sup35 foci that occur as the cells are cured by Hsp104 overexpression. The dissolution model predicts that there will be a loss of seeds due to the trimming activity of Hsp104, whereas the asymmetric segregation model predicts that the seeds will be retained in the mother cell. To examine the changes in GFP-labeled Sup35 foci that occur during curing, we used the L2888 [*PSI^+^*] strain, which has an identical background as the L1758 strain, except that it expresses GFP-labeled Sup35 rather than unlabeled Sup35 (NMC) from the *SUP35* chromosomal locus. 

**Figure 5:**
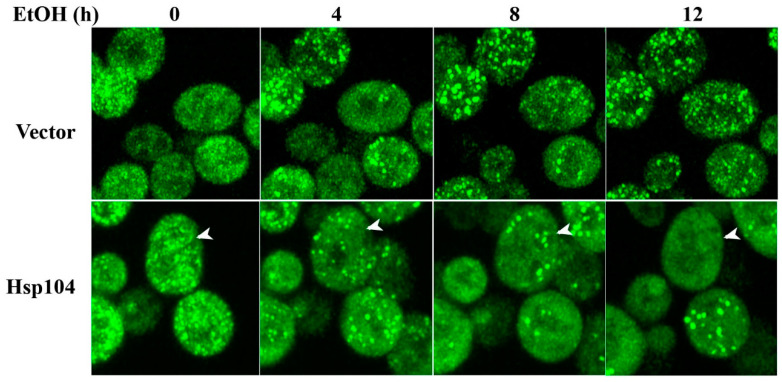
Maximized Z-sack confocal images of GFP-labeled Sup35 in L2888 [*PSI^+^*] strain grown overnight in synthetic galactose medium and 10% ethanol. L2888 [*PSI^+^*] strain was transformed either with vector control or plasmid to overexpress Hsp104. Z-stack confocal images were obtained every 15 min of the same field of cells. In the top panel, the yeast were transformed with the vector control and the bottom panel, the yeast were transformed to overexpress Hsp104 from the *GAL1* promoter. The arrow points to a cell in which there is a loss of detectable foci in the absence of cell division.

Since the L1758 [*PSI^+^*] strain was cured without cell division by expression of high levels of Hsp104 in 10% ethanol, we first imaged the L2888 [*PSI^+^*] strain transformed with the empty vector in galactose and 10% ethanol. As shown in Figure 5 (top row), overnight incubation in ethanol caused an increase in the intensity of the GFP-labeled Sup35 foci, which is consistent with our previous observation that stress increased the brightness of the foci [19,26,31]. However, even after 12 h in 10% ethanol, the foci persisted, and the plating assay showed no curing. Evidently, the expression level of Hsp104 induced by 10% ethanol is not sufficient to cure the L2888 [*PSI^+^*] strain. These results are consistent with our previous studies that showed that the L1758 [*PSI^+^*] strain cures more readily than the L2888 [*PSI^+^*] strain and higher level of Hsp104 expression is needed to cure stronger [*PSI^+^*] variants [26].

To achieve curing of the L2888 [*PSI^+^*] strain, we transformed the L2888 [*PSI^+^*] strain with a plasmid to overexpress Hsp104 from the *GAL1* promoter. When Hsp104 is overexpressed from the *GAL1* promoter for extended times, there is greater than 10-fold Hsp104 overexpression [19]. Galactose and 10% ethanol were then added to this strain to overexpress Hsp104 and simultaneously arrest cell division. Overnight imaging of the same field of cells (Figure 5, bottom row) showed a decrease in the number of foci per cell and some cells showed a complete loss of foci even though there was no cell division (see arrow). This decrease is shown in Table 1, which gives the average foci number per cell in yeast overexpressing Hsp104 in 10% ethanol at different times after addition of galactose and ethanol. The average foci per cell at each time point was calculated by counting the foci number per cell from the multiple focal planes that were imaged for each Z-stack. Interestingly, even though the cells clearly show a loss of foci with time, there is heterogeneity in the seed number per cell in the population. This is consistent with some cells showing rapid curing, while other cells requiring many more generations to cure (see Figure 1B).

When the cells were retrieved from the chamber slide after overnight imaging, we found that they grew a little more than one generation and during that time, the plating assay showed that 7% of the cells were cured and 44% of the cells were red/white sectored, indicating that the yeasts were cured shortly after being plated. Importantly, none of the cells that we imaged showed formation of large aggregates or tethering of the foci to a subcellular structure, mechanisms that have been proposed to account for asymmetric segregation of the seeds by causing their retention in the mother cell. Therefore, the imaging data showing a loss of foci in the absence of cell division are consistent with the dissolution model rather than the asymmetric segregation model for the curing of [*PSI^+^*] by Hsp104 overexpression. 

### 2.5. Curing of [PSI^+^] by Hsp104 Overexpression Using Different Inhibitors to Block Cell Division

To further confirm that the curing of [*PSI^+^*] by Hsp104 overexpression can occur in the absence of cell division, we applied other methods that have been reported to inhibit cell division. Addition of nocodazole was not effective in inhibiting cell division, but we were more successful when we added the mating pheromone, alpha-factor [42]. Based on the optical density measurements, alpha-factor caused only a 30% reduction in growth, but this is likely an underestimation because alpha-factor causes the yeast to form a shmoo-like shape, which we confirmed via imaging. This, in turn, causes the yeast to become much larger. In contrast to ethanol, alpha-factor alone did not cure the L1758 [*PSI^+^*] strain in the absence of Hsp104 overexpression. However, when Hsp104 was overexpressed from the *GAL1* promoter for 4 h in L1758 [*PSI^+^*], there was ~65% curing of the L1758 [*PSI^+^*] strain by Hsp104 overexpression. Since alpha-factor confers at least partial inhibition of growth, it would be expected to affect the extent of curing according to the asymmetric segregation model. However, it did not affect the curing of L1758 [*PSI^+^*] yeast by Hsp104 overexpression indicating that this curing is due to the dissolution of the prion seeds. 

**Figure 6:**
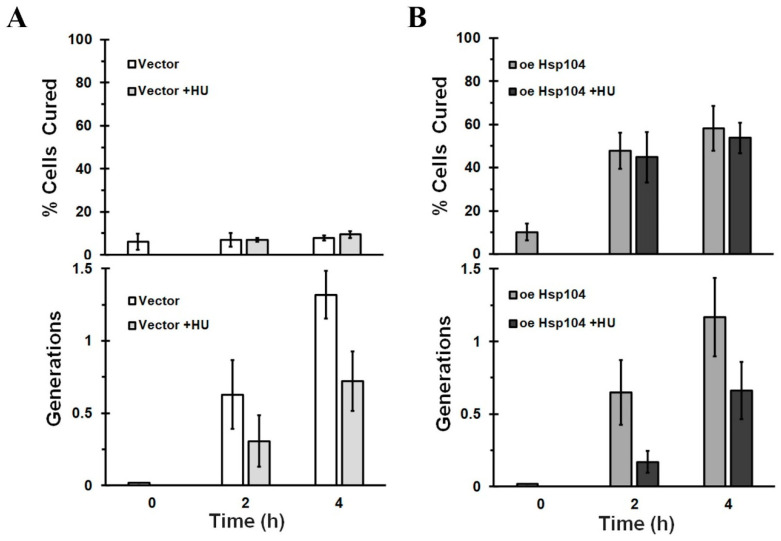
Effect of 0.2 M hydroxyurea (HU) on the growth and the curing of L1758 [*PSI^+^*] yeast by Hsp104 overexpression. L1758 [*PSI^+^*] yeast were transformed with either empty vector (**A**) or *HSP104* on the *GAL1* promoter (**B**). The curing and growth were measured after addition of galactose for either 2 h or 4 h in the presence and absence of 0.2 M hydroxyurea. In the top panels, the percent cured cells are plotted as a function of incubation time and in the bottom panels, the generations are plotted as a function of incubation time. When cells were treated with hydroxyurea, there was a 1 h preincubation time before addition of galactose medium.

We also tried using hydroxyurea [42], which inhibits DNA synthesis and yeast growth to block cell division [43]. Addition of hydroxyurea caused a 50% reduction in growth based on optical density regardless of Hsp104 overexpression (Figure 6 bottom panels), but like alpha-factor, this may be an underestimation of the reduction in yeast growth since hydroxyurea makes the bud size larger [44]. Similar to alpha-factor, but in contrast to 10% ethanol, hydroxyurea did not cure the L1758 [*PSI^+^*] in the absence of Hsp104 overexpression (Figure 6A, top panel). Furthermore, hydroxyurea did not affect the curing of L1758 [*PSI^+^*] by Hsp104 overexpression (Figure 6B, top panel). Specifically, overexpression of Hsp104 for 2 h cured ~50% curing of the L1758 [*PSI^+^*] during which time, the yeast grew 0.3 and 0.6 generations in the presence and absence of hydroxyurea, respectively (Figure 6B, bottom panel). With 0.3 generations of growth, the asymmetric segregation model predicts that there should be less than 20% curing of the [*PSI^+^*] yeast; as pointed out previously, according to the asymmetric segregation model, a minimum of one generation is needed to achieve 50% curing. Therefore, by using hydroxyurea in addition to using 10% ethanol to inhibit cell division, we find that our results are not compatible with the asymmetric segregation model of curing. 

## 3. Discussion

Two different models have been proposed to explain the curing of [*PSI^+^*] by Hsp104 overexpression [21]: the dissolution model in which the trimming activity of the overexpressed Hsp104 causes [*PSI^+^*] curing by reducing the size of the seeds [19,26] and the asymmetric segregation model that attributes [*PSI^+^*] curing to the greater retention of seeds in the mother than the daughter cells [20]. Importantly, these models make very different predictions regarding the curing of [*PSI^+^*] by Hsp104 overexpression. For instance, only the dissolution model first predicts that the mother and daughter cells should cure at the same rate and second predicts that the curing of [*PSI^+^*] should not be dependent on cell division. Given these key differences between the models, we have reexamined the curing of [*PSI^+^*] by Hsp104 overexpression using the L1758 [*PSI^+^*] strain, which is a weaker [*PSI^+^*] variant than the one used in our previous study [19]. The advantage of using a weaker [*PSI^+^*] variant is that the curing of [*PSI^+^*] by Hsp104 overexpression occurs both at a faster rate and at a lower level of Hsp104 overexpression [26].

The results of this study are not compatible with the predictions of the asymmetric segregation model of [*PSI^+^*] curing, which we tested using several different methods. When we used flow cytometry to separate the mother and daughter cells, we found no significant difference in the extent of curing between these populations of cells when the yeast were ~50% cured by overexpression of Hsp104 for one generation. In addition, the rate of curing of [*PSI^+^*] by Hsp104 overexpression was not dependent on Sir2 expression, a protein that is integral to the formation of the polarisome [45]; the polarisome functions in the segregation and retrograde transport of protein aggregates from the daughter cell to the mother cell [33,34]. In contrast to our results, which we obtained in yeasts with two different backgrounds and with different [*PSI^+^*] variants, the Chernoff laboratory reported that deleting *SIR2* reduced the rate of [*PSI^+^*] curing by Hsp104 overexpression [28]. We were unable to determine why our results differed in this regard from those of the Chernoff laboratory.

In contrast to our proposal of the dissolution model, the Tuite laboratory explained the curing of [*PSI^+^*] by Hsp104 overexpression with the asymmetric segregation model. Their evidence for this model came from separating mother–daughter pairs in partially cured yeast, followed by counting the number of prion seeds in these cells [20]. They used a stronger [*PSI^+^*] variant than the one we used in the current study, but it was the same strong variant we used in our previous study where we found no significant difference in the extent of curing between the mother and daughter cells at 3 generations when the yeasts were about 30% cured [19]. Consistent with these results, the Tuite laboratory also did not find a significant difference in the seed number between the mother and daughter cells at 3.7 generations when 30% of the yeasts were cured. Therefore, our laboratories are in agreement that asymmetric segregation of the prion seeds is not evident during the initial phase of the [*PSI^+^*] curing caused by Hsp104 overexpression. 

The Tuite laboratory also measured the number of prion seeds in the mother and daughter cells at 5.7 generations when 50% of the yeast were cured. At this later time point, they found that about three-quarters (43/57) of the mother–daughter pairs showed no significant difference in prion seed number [20]. In fact, they found that the daughter cells, but not the mother cells, were cured in only one-seventh (8/57) of the mother–daughter pairs. Therefore, the data from the Tuite laboratory showed that asymmetric segregation of the seeds only occurred in a small minority of the population at the tail-end of the curing curve, a region where we observed a flattening out of the curing curve. Furthermore, since they did not find a significant difference in the size of the prion seeds in yeast overexpressing Hsp104, it is not clear what is causing greater retention of seeds in the mother than in the daughter cells. Therefore, the asymmetric segregation model does not explain how the majority of the [*PSI^+^*] cells are cured by Hsp104 overexpression. 

Not only did we find no difference in the extent of curing in mother and daughter cells when cell growth was arrested with either hydroxyurea or ethanol, but, in addition, we also we obtained much more curing of the L1758 [*PSI^+^*] strain than predicted by the asymmetric segregation model. In ethanol, we effectively completely arrested cell division and still were able to cure the L1758 [*PSI^+^*] strain. As a control, since the curing of the [URE3] yeast prion by overexpression of Btn2 has been reported to be due to asymmetric segregation of the [URE3] seeds [46,47], we examined whether inhibiting cell division by 10% ethanol would, in fact, prevent this curing. As expected, we found that this was the case; [URE3] was not cured by overexpression of Btn2 when the yeast were incubated overnight in 10% ethanol, whereas in the absence of ethanol, ~50% of the cells were cured. Therefore, inhibiting cell division blocks curing of a yeast prion that is cured by asymmetric segregation in contrast to what occurs when a yeast prion is cured by dissolution where inhibiting cell division has no effect.

Unexpectedly, we also observed that it was not necessary to overexpress Hsp104 to cure the L1758 [*PSI^+^*] strain in ethanol. Ethanol stress has been shown to both arrest cell growth and increase Hsp104 expression from the *HSP104* promoter [32,37], which is consistent with our data. It has also been shown to decrease the efficiency of read-through at stop codons of [*PSI^+^*] yeast, indicating ethanol stress increases the amount of soluble Sup35 [39]. However, in contrast to the current results, this was only a transient effect and once the ethanol was removed, the amount of read-through returned to the pre-stress levels. Although we observed that addition of ethanol cured the weak L1758 [*PSI^+^*] variant, it did not cure stronger [*PSI^+^*] variants; e.g., L2888 [*PSI^+^*]. Furthermore, our results showing that ethanol alone cured the weak L1758 [*PSI^+^*] variant might explain why there is such a low frequency of [*PSI^+^*] in wild-type strains [48,49]; the ethanol produced during fermentation might increase the expression of Hsp104, which then cures the weak [*PSI^+^*] variants that spontaneously form in yeast. 

We also imaged GFP-labeldSup35 foci in [*PSI^+^*] cells overexpressing Hsp104 in 10% ethanol. The images showed a progressive loss of foci and a complete loss of foci in some cells without cell division. These results are not consistent with the asymmetric segregation model for curing since the seeds did not form large aggregates and were freely mobile with no anchoring to a subcellular structure. The loss of foci independent of cell division is consistent with our previous observation that Hsp104 causes a loss of prion seeds due to the trimming activity of Hsp104, which ultimately produces curing when all of the seeds are eliminated by trimming [19]. 

Another stress that cures [*PSI^+^*] is heat shock [28,50,51], which likewise has been shown to increase Hsp104 expression [37]. However, in contrast to the extensive curing observed when yeast were stressed in 10% ethanol, the Chernoff and Serio laboratories have shown that directly after heat shock, only a small percentage of colonies were cured, but many colonies were red/white sectored, indicating that they were cured shortly after plating. Both laboratories suggested that heat shock cures [*PSI^+^*] by asymmetric segregation of the prion seeds, a suggestion that was supported by their finding that deleting proteins which were essential for polarisome formation markedly inhibited curing. However, the findings of the laboratories differ in that the Chernoff laboratory reported that the daughter cells were preferentially cured relative to the mother cells whereas the Serio laboratory reported the opposite; i.e., that the mother cells were preferentially cured relative to the daughter cells. Furthermore, the Chernoff laboratory proposed that the asymmetric segregation of the prion seeds was due to the sequestering of the aggregated prion seeds in the mother cell, whereas the Serio laboratory proposed that the asymmetric segregation was due to the presence of higher levels of Hsp104 in the mother cells than in the daughter cells. Despite the differences between these laboratories, their results make clear that heat shock causes [*PSI^+^*] curing by a very different mechanism than ethanol stress. Therefore, one cannot generalize that because heat shock causes [*PSI^+^*] curing by the mechanism of asymmetric segregation of the prion seeds, this is also the case for other conditions where Hsp104 is overexpressed; e.g., ethanol stress. Rather, our results clearly show that ethanol stress causes [*PSI^+^*] curing by the dissolution mechanism; i.e., curing is caused by the trimming activity of the highly expressed Hsp104. 

It has been proposed that Hsp104 has the therapeutic potential to protect higher eukaryotes against the toxic effects of neurodegenerative disease substrates, such as TDP-43, FUS, and α-synuclein [52,53,54]. The Shorter laboratory found that, in particular the N-terminal domain of Hsp104 is necessary to mitigate the toxicity caused by TDP-43 or α-synuclein in yeast cells [53]. Interestingly, the Masison laboratory found that the curing of [*PSI^+^*] by Hsp104 overexpression was also dependent on the N-terminal domain of Hsp104, indicating that this domain functions in the trimming of the prion seeds by Hsp104 [55]. This, in turn, suggests that the trimming activity of Hsp104 may be useful in reducing the toxicity of substrates that cause neurodegenerative disease in higher eukaryotes. 

## 4. Methods

### 4.1. Yeast Strains, Plasmids, Culture Conditions, and Plating Assay

The L1758 and L2888 yeast strains used in this paper are derivatives of 74D-694 (*MATa ade1*-*14*, *trp1*-*289 his3*-*Δ200 ura3*-*52 leu2*-*3,112*) [56]. The L2888 strain expresses GFP-labeled Sup35, which was constructed by inserting the GFP between the N-terminal and M-terminal domains of Sup35. Other strains used in this study were the 779-6A yeast strain (*MATa, kar1-1, SUQ5, ade2-1, his3Δ202, leu2Δ1, trp1Δ63, ura3-52*) [16] and the 1660 yeast strain (*MAT*α*, kar1-1, P_DAL5_∷ADE2, his3*Δ*202, leu2*Δ*1, trp1*Δ*63, ura3-52, P_RNQ1_∷URE2-GFP∷rnq1.)* [47].

To delete *SIR2*, the *sir2::KanMX4* allele was PCR-amplified from the strain BY4742 using primers 455 bp upstream of the ATG initiator codon (-455) and 252 bp downstream of the TAA stop codon (+252). The 2.4 kb product was used to transform strain 779-6A and L1758 (strain1661) selecting on YPAD containing 300 mg/L G418. Resulting G418-resistant candidates were confirmed to integrate at the correct locus using primer pairs *SIR2* -488 (forward)/KanB (reverse), and KanC (forward)/*SIR2* +311 (reverse). Strains used were selected after backcrosses to wild type. Deletion of *STI1* was conducted as described previously [27]. 

The plasmids used in these experiments were the pRS314 empty vector, the pRS314 vector with *HSP104* on the *GAL1* promoter [19], or pRS315-*BTN2* on the *GAL1* promoter [47]. 

Yeasts were grown at 30 °C on synthetic defined medium (SD, 0.7% yeast nitrogen base, 2% glucose) with complete supplement mixture or the appropriate amino acid dropout for selection and maintenance of the plasmid (Sunrise Scientific). Synthetic galactose (SGal) medium contains both 2% galactose and 2% raffinose in place of glucose. ½ YPD solid medium used in the plating assays contains 0.5% yeast extract, 2% peptone, and 2% glucose. Cultures were always maintained in active growing conditions (OD_600_ ≤ 0.6) by periodic dilution with fresh medium. When cells were cured in galactose medium, they were grown overnight in raffinose selection medium prior to the addition of galactose selection medium. Growth was monitored by absorbance at 600 nm and curing was monitored by plating the yeast on ½ YPD plates. Colonies with any white sectors originating from a [*PSI^+^*] yeast were counted as [*PSI^+^*]. A minimum of three colonies were averaged for each experiment. 

### 4.2. Methods for Arresting Cell Division

Cells were kept in the logarithmic phase (OD 600 < 0.6) for 24 h prior to experiment and were grown in a 30 °C incubator. At the start of the experiment, cells were diluted to an OD 600 of 0.20. To inhibit cell division, we used 15 µg/mL of nocodazole (Sigma, St. Louis, MO, USA), 5 µg/mL alpha-factor (Zymo Research, Irvine, CA, USA) or 0.2 M hydroxyurea (Sigma). To arrest with ethanol, 100% ethanol was added to the cells to make a final concentration of 10% ethanol. Yeast growth was also monitored by measuring the absorbance of the samples at 600 nm using the Synergy H1 microplate reader (Biotek). Yeast in a 48-well tissue culture plates (Sarstedt) were maintained with constant shaking at 30 °C. Measurements were obtained at 10 min intervals for 24 h. 

### 4.3. Flow Cytometry of Stained Yeast

L1758 [*PSI^+^*] yeasts were kept in the logarithmic phase (OD 600 < 0.6) for 24 h prior to experiment and were grown at 30 °C. For staining, fluorescent disodium 28 brightener (Sigma) was added to the cells at a final concentration of 0.005% for 5 min [57]. After 5 min, the disodium brightener was washed out of the cells and the cells were resuspended in selection medium and grown for 4 h at 30 °C. The Becton Dickerson FACSAria Fusion flow cytometer was used to separate and collect the fluorescent-stained mother cells and the non-fluorescent daughter cells. Cells were plated both before and after the yeast were separated via flow cytometry.

### 4.4. Confocal Microscopy

Fluorescence images were obtained on the Zeiss LSM 880 Airyscan confocal microscope equipped with a 63× 1.4 NA objective. Z-stack confocal images were taken for all cells. The yeasts were imaged in 8-well 25-mm^2^ chambered coverslips (Cellvis).

### 4.5. Western Blots

Cell lysis and Western blotting were performed as described previously [31] The following antibodies were used: a polyclonal rabbit anti-Hsp104 antibody (Thermo Scientific Project NJ1240S), anti-Pgk1 monoclonal antibody (Molecular Probes, Carlsbad, CA, USA), and rabbit anti-Hsp70 antibody (Enzo SPA 757). HPR conjugated goat anti-rabbit or goat anti-mouse secondary antibodies (Thermo Fisher, Waltham, MA, USA) and SuperSignal Plus Chemiluminescent Substrate (Thermo Scientific) were used to develop the Western blot. Amersham Imager 600 was used to scan the blot and the intensities were quantified using the ImageJ-win64.

## Figures and Tables

**Table 1:** Quantification of average foci number per cell as a function of time.

Time (h)	Avg Foci No Per Cell	St Dev
0	>100	ND
4	57	23
8	22	20
12	7	15

Quantification of foci/cell were obtained by analyzing the multiple focal planes that were acquired in the Z-stacks of the L2888 [*PSI^+^*] cells overexpressing Hsp104 in 10% ethanol. The data are an average of foci number per cell obtained from 15 cells that remained in the imaging field without dividing.

## Data Availability

The data supporting the findings of this is available on the following website: https://nhlbi.figshare.com/articles/dataset/Data_for_paper_on_High_Expression_Levels_of_Hsp104_Cures_the_Yeast_em_PSI_em_Prion_by_Dissolution_of_the_Prion_Seeds/23059844 (posted on 20 June 2023).
